# The Role of Macrophage/B-Cell Interactions in the Pathophysiology of B-Cell Lymphomas

**DOI:** 10.3389/fonc.2018.00147

**Published:** 2018-05-08

**Authors:** Lan V. Pham, Elizabeth Pogue, Richard J. Ford

**Affiliations:** Department of Hematopathology, The University of Texas MD Anderson Cancer Center, Houston, TX, United States

**Keywords:** B-cell lymphoma, lymphoma-associated macrophages, tumor microenvironment, immune suppression mechanism, macrophages

## Abstract

Macrophages (MPs) are heterogeneous, multifunctional, myeloid-derived leukocytes that are part of the innate immune system, playing wide-ranging critical roles in basic biological activities, including maintenance of tissue homeostasis involving clearance of microbial pathogens. Tumor-associated MPs (TAMs) are MPs with defined specific M2 phenotypes now known to play central roles in the pathophysiology of a wide spectrum of malignant neoplasms. Also, TAMs are often intrinsic cellular components of the essential tumor microenvironment (TME). In concert with lymphoid-lineage B and T cells at various developmental stages, TAMs can mediate enhanced tumor progression, often leading to poor clinical prognosis, at least partly through secretion of chemokines, cytokines, and various active proteases shown to stimulate tumor growth, angiogenesis, metastasis, and immunosuppression. Researchers recently showed that TAMs express certain key checkpoint-associated proteins [e.g., programmed cell death protein 1 (PD-1), programmed cell death-ligand 1 (PD-L1)] that appear to be involved in T-cell activation and that these proteins are targets of other specific checkpoint-blocking immunotherapies (anti-PD-1/PD-L1) currently part of new therapeutic paradigms for chemotherapy-resistant neoplasms. Although much is known about the wide spectrum and flexibility of MPs under many normal and neoplastic conditions, relatively little is known about the increasingly important interactions between MPs and B-lymphoid cells, particularly in the TME in patients with aggressive B-cell non-Hodgkin lymphoma (NHL-B). Normal and neoplastic lymphoid and myeloid cell/MP lineages appear to share many primitive cellular characteristics as well as transcriptional factor interactions in human and animal ontogenic studies. Such cells are capable of ectopic transcription factor-induced lineage reprogramming or transdifferentiation from early myeloid/monocytic lineages to later induce B-cell lymphomagenesis in experimental *in vivo* murine systems. Close cellular interactions between endogenous clonal neoplastic B cells and related aberrant myeloid precursor cells/MPs appear to be important interactive components of aggressive NHL-B that we discuss herein in the larger context of the putative role of B-cell/MP cellular lineage interactions involved in NHL-B pathophysiology during ensuing lymphoma development.

## Introduction

Macrophages (MPs) are intrinsic end-stage immune myeloid cells that contribute to homeostasis as well as the body’s natural cellular immune and long-term inflammatory responses. As functional immune effector cells, they also serve as important links between the so-called intrinsic and adaptive immune systems that contribute to a biochemical milieu involving complex inflammatory events (1, 2).

Several subtypes of activated MPs exist, and they contribute differently to the immune microenvironment (3, 4). Specifically, MPs differ in their responses to signals from both the adaptive and innate immune systems, with various potential outcomes. Whereas the subtypes of MPs vary extensively, representing highly attenuated responses to different immune cellular events, the opposing forces of classic vs. alternative cellularly activated MPs are of particular interest. Induced by exposure to interferon-γ in the presence of various bacterial microbes, classically activated MPs (M1 MPs) produce various activated lytic enzymes, including interferon-α, and reactive oxygen species in addition to inflammation-promoting chemokines (5). In short, these MPs create a hostile microbicidal environment. In contrast, alternatively activated MPs (M2 MPs) repair tissues using molecules such as growth factors and transforming growth factor-β to reduce inflammation *via* molecules such as interleukin (IL)-10 and transforming growth factor-β. In the normal immune system, the different subtypes of MPs induce distinct types of immune responses to various antigens, specifically, viral and bacterial antigens (M1 MPs) and parasitic as well as fungal antigens (M2 MPs). The interplay between M1 and M2 MPs exists on a continuum. It can both resolve inflammation and, as in tumor microenvironments (TMEs), minimize inflammation and immune surveillance while increasing life expectancy (6).

Tumor-associated MPs (TAMs) are components of a highly complex and heterogeneous TME of productive host cells (7, 8). For example, specific TME signatures of lymphomas can aid in the maintenance of neoplastic cells experimentally *in vitro* and probably *in vivo*. The microenvironment’s impact on cell growth and destruction varies greatly according to the inherent histotype of the lymphoma cell type. For example, the Hodgkin lymphoma (HL) tissue often consists of relatively few monoclonal cancer cells but at least 90% non-malignant cells (e.g., regulatory T cells), contributing to a fairly unique surrounding TME ecosystem (9), whereas Burkitt lymphoma seems to be largely devoid of a supportive cellular environment (6, 10). Even in cases of Burkitt lymphoma, though, immune signaling and attenuation (i.e., by IL-10) are crucial to intrinsic lymphoma cell proliferation (11). Clearly, TAMs play a distinct, specific, important role in neoplastic progression.

The presence of MPs in a tumor can be indicative of several characteristics of a lymphoma’s clinical signature, including prognosis as well as efficacy of chemotherapy (12). Even before cells become cancerous, MPs can add to their surrounding inflammatory environment, producing mutagenic substances like reactive oxygen species that may support or augment oncogenesis (13). In addition, M2 MPs can express key immune checkpoint molecules, including programmed cell death protein 1 (PD-1) and programmed cell death-ligand 1 (PD-L1), generally inhibiting the overall inflammatory response, allowing the tumor cells to evade antitumor immunity (14).

Macrophages exist in complex TMEs and interact with other cells therein. In particular, interaction between lymphocytes and MPs may create a hostile tumorigenic intrinsic environment. For example, fibrosis in follicular lymphoma (FL) cases is correlated with the presence of both Th2 T cells and their related M2 MPs (15). In these cases, MPs and fibroblasts both contribute to fibrosis. In addition, CD8+ T-cell infiltrates and MPs are found at higher numbers in higher Ann Arbor-stage lymphomas, suggesting an association between not only MPs and cellular tumor extent but also between T cells and MPs in advanced B-cell lymphomas (16). Furthermore, communication between lymphocytes and MPs is diverse and variable. T and B cells both interact with TAMs in ways that can impact the progression of lymphoma and its clinical response to chemotherapy. Whether TAMs suppress an active immune response or impact the efficacy of immune therapy, they represent crucial junctions between innate and/or acquired immune systems that should not be overlooked or underappreciated in the pathological processes involved in hematologic malignancies.

## B-Cell/MP Interactions in B-Cell Lymphomas

### History

One of the first descriptions of the interactions between MPs and malignant B cells came in the 1960s with a number of studies in which researchers employed electron microscopy with ultrastructural pathological staining to identify MPs in lymphomas (17, 18). The most common description of lymphoma-associated MPs was the classic “starry sky” histological appearance in Burkitt lymphoma, in which the tumor cells have a very high turnover rate, so TAMs phagocytose and scavenge the tumor and stuff it with cytoplasmic cellular debris (at this point, the TAMs are called tingible body MPs). Upon fixation, the cytoplasm in TAM retracts, leaving round white spaces filled with debris resembling stars (19, 20). This pattern can be seen in both high-powered paraffin-embedded bone marrow and lymph node sections characteristic of Burkitt lymphoma (21, 22).

In the 1970s, investigators in several studies used a modified “skin window” (glass coverslip) technique to study MPs in normal subjects and patients with HLs or non-HLs (NHLs) (23, 24). They discovered that large MP-like cells in these patients were morphologically abnormal, exhibiting multinucleated patterns and, in some cases, aberrant multipolar mitotic figures. They concluded that the presence of abnormal MPs in lymphomas may be related to a significant and aggressive malignant process, but follow-up research of these findings was either lacking or inconclusive. In fact, several authors initially described these large MP-like cells both generically and descriptively as diffuse large cell lymphoma cells exhibiting considerable morphological plasticity and as dominant types of large cell in primitive lymphoreticular-type neoplasms (25). For many years, researchers generically described such large atypical (undifferentiated) lymphoma cells in terms of their large size and irregular shape (atypia) as abnormal lymphoid tumor cells of obscure genetic cellular origin or as generic primitive lymphoid tumor cells, which were then referred to as reticulum cell sarcoma cells in the original pre-Rappaport early hematological fascicle of lymphoid classification terminology (26). These diffuse large cell lymphoma cells were later better characterized pathologically and subsequently designated as histiocytic lymphoma cells by Rappaport in his classic NHL/World Health Organization histopathological classification monograph. Primarily using light microscopy, clinical hematological researchers later showed that most of these cells were large, atypical polymorphous/polyploid lymphoid tumor cells categorized as various large lymphoid cell types, such as diffuse, undifferentiated, and/or immunoblastic histotypes, including occasional rare, possibly true histiocytic tumor cell types. However, at least 85% of these generically described diffuse large cell lymphomas are now shown to be derived from a clonally transformed neoplastic B-lymphoid lineage [diffuse large B-cell lymphoma (DLBCL)] without definitive evidence of a “true” or validated histiocytic cellular origin or lineage (27). In more recent and potentially more convincing follow-up studies originally conceptualized and performed by Lukes and Collins (28), they identified morphologically diffuse large cell lymphoma cells as actually being neoplastically transformed large, atypical B-lymphoid cells possibly of early B-cell lineage rather than precursor cells of the previously believed early myeloid/monocytic-derived or malignant histiocyte/MP origin originally conceived and favored by Rappaport and colleagues in their early World Health Organization classification.

In the early 1980s, several *in vitro* models of lymphoma-derived MPs were described (24, 29). In one study, pleural effusions from patients with diffuse “histiocytic” lymphoma (currently known as DLBCL) were cultured *in vitro*, giving rise to an MP population with a wide variety of cellular functions, including promotion of lymphoma cell growth and survival, as well as inhibition of immune responsiveness. These studies also demonstrated that lymphoma-derived MPs differed markedly from normal peripheral blood donor-derived MPs, as the former could, for instance, induce formation of lymphocytic rosettes around tumor MPs. However, the functional significance of lymphocytic rosette formation was difficult to explain at that time, requiring additional probing into mechanisms that could underlie this phenomenon. Such findings may have provided an initial clue or indication regarding how indigenous tumor-derived MPs interact and communicate with B-cell lymphoma cells and identified that such interactions could have important clinical as well as biological implications (30, 31).

Not until the early 2000s did authors begin to report on studies demonstrating that infiltrating MPs within lymphomas promote their biological and/or clinical progression. Researchers in those studies primarily used key phenotypic markers such as CD68 and CD163 to detect TAMs, correlating marker expression patterns with clinical outcome in patients with different types of lymphoma (32, 33).

### TAMs in HL Patients

Investigators first described the clinically significant role of TAMs in HL cases in 1985 when they used peanut agglutinin biomarker staining of paraffin-embedded sections of HLs to identify MP histioctyes. They demonstrated that increased numbers of MP histioctyes in the HL sections correlated with unfavorable clinical and pathological parameters of the disease (34). In 2010, Steidl et al. (35, 36) used gene expression profiling to identify a TAM gene signature that was significantly associated with primary treatment failure. They further validated these findings using CD68 immunostaining, showing that an increased number of CD68+ TAMs in HL patients’ lymph node biopsy samples was associated with adverse clinical prognosis. Since then, a large number of studies have further validated the important correlation between increased numbers of TAMs according to CD68 expression and a poor clinical course of HL (37–40). In fact, researchers identified CD68+ TAMs in relapsed/refractory HL samples, although additional clinical validation is probably required to confirm a role for these biomarkers as adverse prognostic markers of HL (37). However, several studies did not demonstrate a significant correlation between CD68+ TAMs and clinical prognosis for HL (41). Several reasons for these discrepancies are possible, including the background characteristics of the studied patient population, the antibodies and immunostaining reagents and technical methods used, and the numerical scoring methods used and analyzed.

Besides CD68, additional TAM biomarkers, such as the similarly expressed CD163 and colony-stimulating factor 1 receptor (CSF-1R) diagnostic antibody-based reagents, may be useful to stratify prognosis for HL (40, 42). Expression of CD163, a specific biomarker of M2 TAMs, seems to be a better, more efficient biomarker than CD68 in predicting clinical outcomes of HL (39). In addition, the lymphocyte/monocyte ratio was associated with the presence of TAMs and identified as a negative prognostic indicator for HL, suggesting another biologic marker that can be used to predict clinical outcome of HL (43).

Overall, these findings are encouraging, as they clearly demonstrated the clinical significance of TAMs regarding HL in various patient cohorts. However, the pathological and biological significance of TAMs in HL patients must be examined and validated. What are the biological functions of TAMs in the setting of HL, in which several cell types, including T cells, B cells, and Reed–Sternberg cells, are involved? Clearly, much more in-depth research in this field is needed to determine whether targeting TAMs in HL patients is feasible or effective in the clinic. Several novel strategies are used to target TAMs, which are described below, but currently, only one known clinical trial is testing treatment of relapsed or refractory HL patients with a CSF-1R inhibitor (44).

### TAMs in FL Patients

Authors have described the clinical significance of TAMs in different subtypes of B-cell lymphomas, mostly as poor prognostic indicators (45–47). However, a few studies demonstrated that the presence of TAMs could indicate a favorable prognosis for these tumors (48, 49). Similar to those for HL described earlier, these discordant results may be attributable to differing methodological approaches as well as the cellular nature of the tissue samples being examined. In addition, the lymphoid cell type and differentiation stage of a B-cell lymphoma may determine the functional significance of TAMs. For instance, in FL patients, the tumor cells are admixed with heterogeneous lymphoid-like stromal cells within infiltrated lymph nodes and bone marrow (50). These stromal cells are involved in the recruitment and polarization of mature monocytes into active M2 MPs. FLs are described as indolent low-grade B-cell lymphomas in which the tumor cells cannot survive on their own, requiring growth and/or survival stimulatory factors from their microenvironments, such as MPs, which appear to maintain the viability of the tumor cells. Various growth factors, such as IL-2, IL-4, IL-15, and CD40L, as well as the immune suppressor cytokine IL-4I1, are known to be secreted by TAMs and are involved in FL pathogenesis (51–53). Clearly, TAMs are biologically linked with FL, presumably providing the proper signals to not only maintain the viability of FL cells but also protect the tumor cells from the active immune system. Based on results of gene expression profiling analysis, investigators found that a defined MP-enriched gene expression pattern was associated with inferior clinical course of FL (54), indicating that the presence of TAMs in FL patients may predict clinical outcomes. In addition, researchers have used other macrophagic markers identified using immunohistochemistry, such as CD68 and CD163, to predict unfavorable outcomes of FL (45–47).

### Our Laboratory Studies and Observations of TAMs in Mantle Cell Lymphoma (MCL) Cases

Mantle cell lymphoma is one of the most challenging human cancers to treat, particularly among hematopoietic neoplasms, and is often one of the most aggressive forms of B-cell non-Hodgkin lymphoma (NHL-B) (55–58). Only relatively recently recognized as a form of NHL-B (1992) (59), MCL was described in the older NHL (1964) literature and in the updated Kiel NHL classification systems as belonging to centrocytic “intermediate” B-cell lymphoma subtypes that are often aggressive, morphologically distinct, small B-cell histotypes of NHL-B (60, 61). MCL was morphologically defined as an aggressive lymphocytic (small cell) lymphoma with scattered epithelioid or “pink” histiocytes (MPs) and referred to as centrocytic intermediate-stage lymphocytic lymphoma. Later, MCL was immunophenotyped as “typical” MCL, the dominant form of the disease, with rarer aggressive blastoid and less aggressive “mantle-zone” variants. Recently, increasing numbers of MCL cases have emerged and been recognized to behave indolently and were associated with longer survival durations than is the classic form of MCL. However, closer scrutiny of these indolent MCL cases suggested that most were non-nodal, were less disseminated than the classic MCL, or tended to be leukemic, exhibiting certain immunophenotypic genotypic characteristics (56). These examples include the so-called *in situ* MCL cases, with or without SOX11 gene expression (62–65). Clearly, MCL is not the mostly monolithic pathological entity that it was previously assumed to be, and the initial indolence of the tumor and presence of pink histiocytes may be important pathophysiological clues, although their overall significance is still unclear. Only a few studies have linked monocyte count with the prognostic impact of MCL (66–69), and studies suggesting functional roles for MPs in MCL are limited. Clearly, active *in vitro* studies are needed for better characterization and biological functions of MPs in MCL biology and pathophysiology.

**Figure 1:**
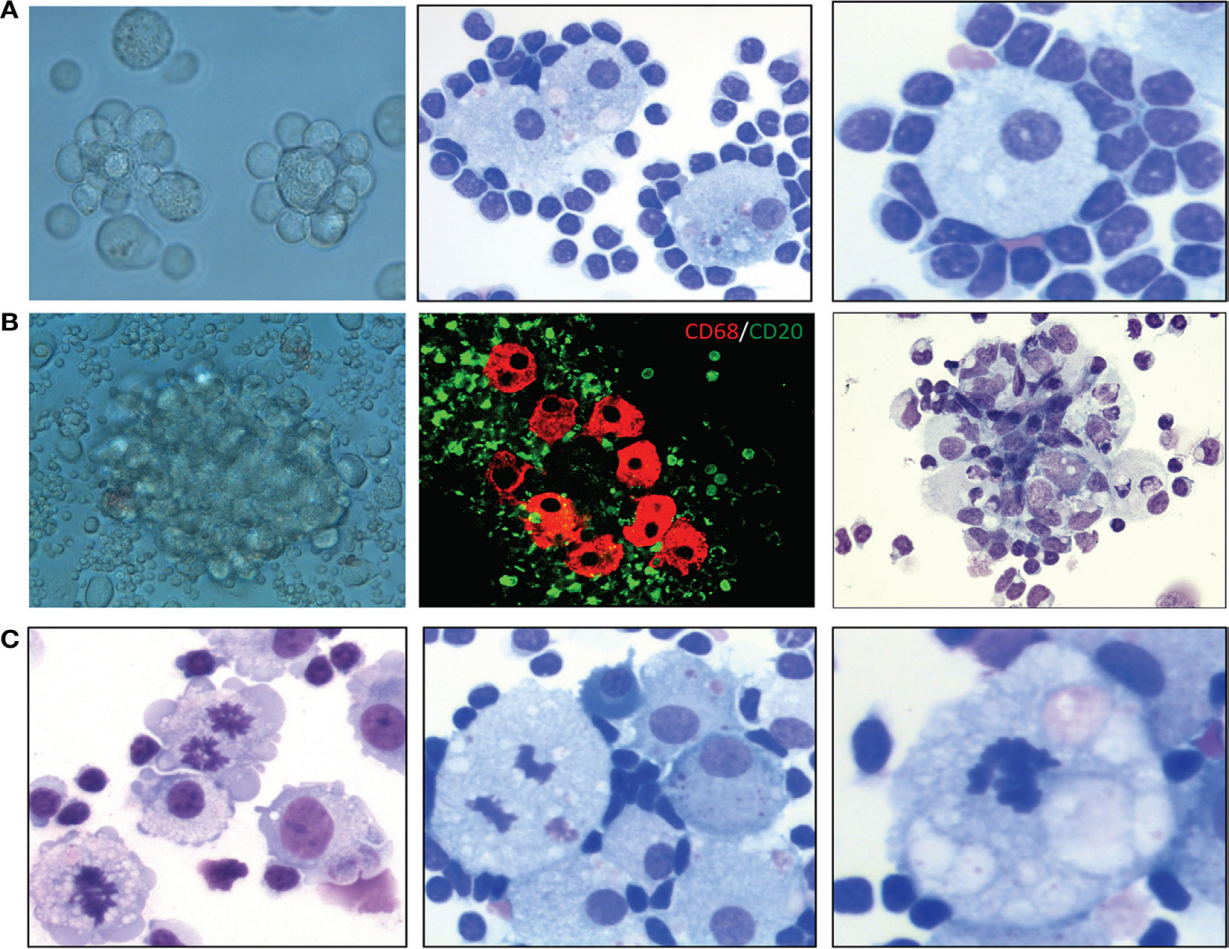
Characterization of lymphoma-associated macrophages (MPs) in B-cell lymphoma cell cultures. **(A)** Examples of lymphoma cell–tumor-associated MP colony aggregation in culture after 2 weeks. Left, phase-contrast light microscopic image; middle, Wright–Giemsa stain (400×); right, Wright–Giemsa stain (400×). **(B)** Examples of MP clustering/aggregation in mantle cell lymphoma (MCL) cell culture under hypoxic conditions. Left, phase-contrast light microscopic image; middle, confocal microscopic analysis of CD68+ MPs (red) and CD5+ cells (green); right, hematoxylin and eosin stain (200×). **(C)** Hematoxylin and eosin and Wright–Giemsa stains showing mitotic figures in MPs in primary MCL culture after 8 weeks under hypoxic conditions (400×).

We recently demonstrated that certain microenvironmental interactions involving cellular subsets of monocyte/MP lineage are necessary for long-term *in vitro* cell culture and pathological characterization of primary MCL cells (70). Primary MCL tumor cells do not spontaneously grow after *in vitro* explanation; they need active cellular interactions with microenvironmental cellular components to stimulate and maintain expanded lymphoma cell growth and survival. Perhaps not surprisingly, monocytic and related cells of mostly myeloid accessory and precursor cell lineages make up a group of “nurse-like” cells from bone marrow and possibly other lymphoid tissues. These cells provide microenvironmental co-factors necessary for maintenance of lymphoma cells *in vitro* and, probably, *in vivo* (71–73). Our recent published studies of large numbers of mostly leukemic/effusion-selected MCL patients demonstrated that when adequate numbers of unstimulated and/or unseparated MCL cells from effusions (>90% morphological) or leukemic cell populations are cultured, the initial result is spontaneous formation of increased numbers of MPs after 7–14 days in cell culture. Furthermore, these MPs stain for CD68 biomarker (70). The MPs are presumably derived from cryptic CD68+ monocytes, as cultures of purified CD20+ lymphoma cells alone usually do not contain CD68+ cells. In addition, treating these cultures with the MP-depleting agent liposomal clodronate (74, 75) completely eliminated these MPs, suggesting that spontaneously formed MPs resemble endogenous TAMs. These TAMs are often bound and encircled by atypical lymphoma B cells (rosettes) *in vitro*, maintaining and nurturing the lymphoma cells in culture for variable durations, usually at least 2 months. The lymphoma cells then often transform into autonomously growing B-cell lymphoma cell lines or slowly die out owing to apparent spontaneous apoptosis. We recently discovered that culturing these primary MCL cells under hypoxic conditions enhances the activation of these TAMs, increasing lymphoma cells’ viability and extending their survival. More importantly, we discovered that culturing primary MCL cells under hypoxic conditions causes them to progressively become adherent rosettes of lymphoma cell–TAM colony aggregates *in situ* (Figure 1A). These predictable clusters or aggregates of lymphoma cells and TAM cells reproducibly form in culture flasks, expanding in size and exhibiting protracted growth and survival (Figure 1B). In some cases, the TAMs frequently exhibit mitotic figures with morphological atypia, indicating that these TAMs are proliferating and may be abnormal (Figure 1C). Our *in vitro* data demonstrated a physical cellular (juxtacrine signaling) relationship between TAMs and lymphoma cells, mimicking the lymphoma cell/MP interactions seen in some bone marrow biopsies in lymphoma patients (76–78).

### TAMs in DLBCL Patients

Diffuse large B-cell lymphoma, the most common human lymphoma, comprises a genetically and clinically diverse group of aggressive NHL-Bs among a small group of important human cancers that have increased in incidence in the United States over the past 4 decades (79–82). Current frontline DLBCL therapy, although fairly successful [~70–80% remission rates with the frontline chemotherapy regimen cyclophosphamide, doxorubicin, vincristine, and prednisone (CHOP) combined with rituximab (R-CHOP)], is frequently followed by relapse (~40% of cases within 2–3 years), often as refractory DLBCL, resulting in only poor salvage therapy responses (<20% partial or complete responses) with short survival durations. Similar to FL, gene expression profiling has identified the TME and host inflammatory response signatures as defining features of DLBCL (83, 84). These studies demonstrated a strong correlation between TME signatures host cells and clinical prognosis for DLBCL. For instance, Lenz et al. (83) reported on a prediction model composed of two TME signatures, stromal-1 and stromal-2, that can predict clinical outcomes in DLBCL patients. Stromal-2 signature genes encoded for well-known markers of monocytic lineages that were predictive of unfavorable survival in DLBCL patients given CHOP alone or R-CHOP. Monocytic myeloid-derived suppressor cells and TAMs are presumably the important cellular types in the stromal-2 signature, as these cells also exhibited prognostic significance for DLBCL in other studies (85–88). Also, a number of studies have shown that a high absolute monocyte count at diagnosis is useful for prognostic stratification of patients with DLBCL (89–92). Khalifa et al. (93) demonstrated that increased CD14+ monocytes with loss of human leukocyte antigen-DR expression were seen in DLBCL patients with higher stage disease, more aggressive pathology, and in relapse or refractoriness to treatment. These studies clearly demonstrated that monocytes play an important role in the pathophysiology of DLBCL, possibly as precursors to TAMs, particularly those with the M2 phenotype. In addition, some studies demonstrated that high expression of CD68 in TAMs correlates with poor prognosis for DLBCL (76, 78, 94), whereas other studies did not demonstrate a specific or significant correlation (95, 96). This discrepancy is probably due to the diagnostic antibodies used in these studies as well as the scoring method used to analyze immunohistochemical CD68 staining. Therefore, double staining for CD68 and CD163 may be a better method of predicting outcomes of DLBCL, as their expression are associated with adverse outcomes in R-CHOP–treated patients (97).

Unlike FL and some MCLs, DLBCL is usually aggressive, with tumor cells that grow more or less autonomously and probably not needing external growth or survival stimuli, at least from TAMs. Given these characteristics, why do TAMs infiltrate or allow recruitment within DLBCL tumor tissues? A possible reason is that DLBCLs must be able to escape the immune surveillance of tumor-specific cytotoxic T cells by recruiting and polarizing M1 TAMs to M2 TAMs that highly express immune checkpoint molecules, such as PD-L1 and PD-L2, on their surfaces (98). These ligands interact with the PD-1 receptor expressed on intratumoral T cells and provide inhibitory signals, thereby suppressing antitumor immune response (99). Immune checkpoint inhibitors, such as anti-PD-1 antibody, bind to the PD-1 expressed on activated cytotoxic T cells, thereby stimulating their proliferative capacity and enabling the immune system to resume recognizing, attacking, and destroying active cancer cells (100, 101). This may be one reason why anti-PD-1/PD-L1 therapy was effective against some cases of DLBCL in a clinical trial (102). Another possibility is that TAMs are recruited to a tumor site to protect the tumor cells from various effects of different chemotherapies. For example, Shen et al. (77) demonstrated that M2 TAMs secrete an enzyme called legumain that promotes the degradation of fibronectin and collagen I, resulting in tumor progression, at least in some murine DLBCL models.

## Origin of TAMs in B-Cell Lymphomas

### Derivation From Cells of Monocytic Lineage

A basic concept regarding the mononuclear phagocytic system is that in most cases, human MPs are derived from myeloid precursor-lineage monocytes (103). Cells of mononuclear phagocyte lineage progress through a series of specific morphologically distinct stages: they possess a myeloid progenitor in common with that of granulocytic cell types giving rise to monoblasts, promonocytes, and, later, monocytes that subsequently migrate into various hematopoietic tissues. In response to infection, monocytes move into focal tissue spaces around sites of infectious involvement and then differentiate into dendritic cells and typical MPs. Although hematological researchers have not formally proven it, many studies have suggested that lymphoma tissue-derived MPs can be derived from circulating monocytes. In fact, a number of studies demonstrated that a high monocyte count ratio at presentation in B-cell lymphoma patients was associated with increased numbers of CD163+ TAMs, which could predict poor clinical outcome (68, 69). The postulated mechanism of this relationship is that a high monocyte count can be a surrogate biomarker for the TME, reflecting the functions of recruited immunosuppressive peripheral blood monocytes recruited by lymphoma cells to differentiate targeted monocytes into polarized MPs (M2) that can in turn activate the tumor cells. Under stressful conditions, such as during *in vitro* cell culture, monocytes may be able to differentiate or be reprogrammed into MPs to provide tumor cells with growth and survival stimulatory factors in an autocrine or paracrine secretory fashion. This is probably the case in MCL patients, in whom pink histiocytes may develop to protect the surrounding tumor cells. A recent study demonstrated that B1 lymphocytes expressing IL-10 and other important chemokines play key roles in recruiting monocytes and promoting a protumoral M2 phenotype of MPs (104). The investigators indicated that *in vivo* polarization of MPs by B1 cells (normal counterparts of MCL cells) likely occurs only in specific anatomical compartments (the peritoneum and spleen) where B1 cells and MPs co-exist.

### MP/Tumor Cell Fusion

**Figure 2:**
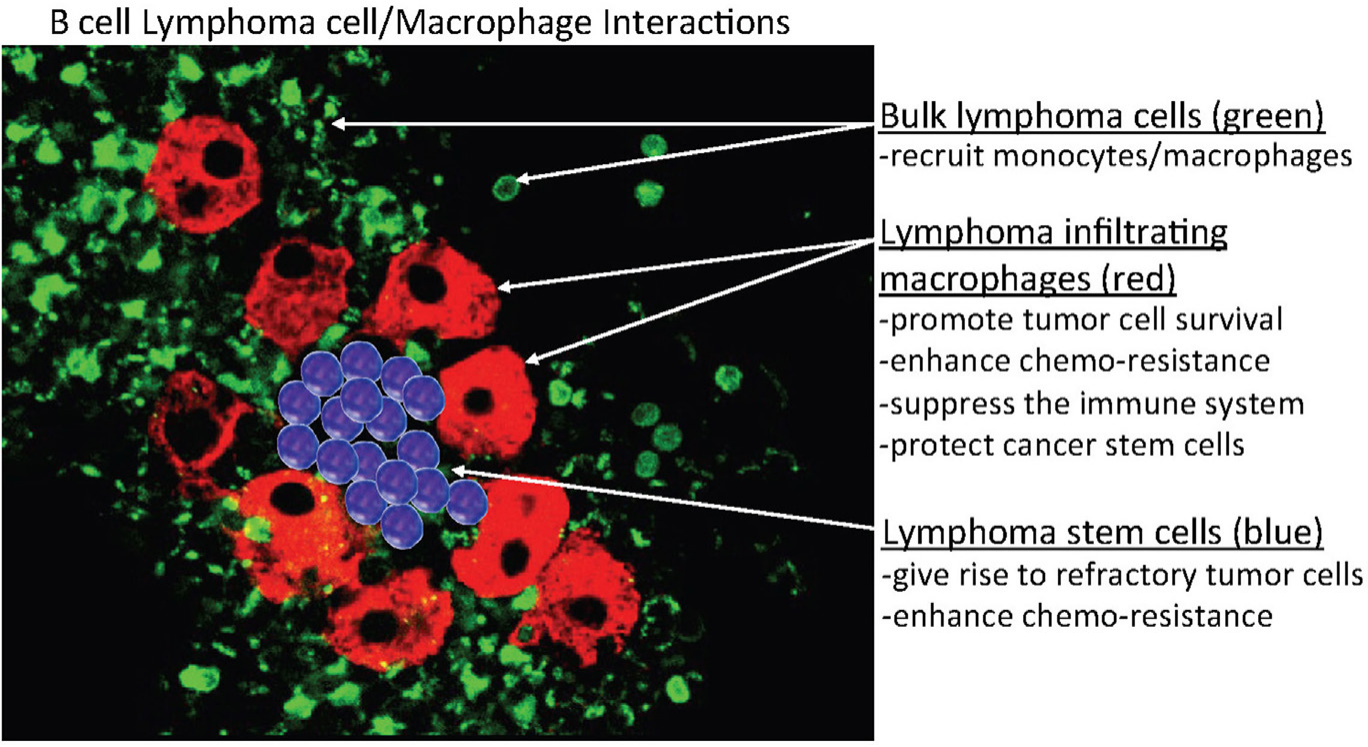
Hypothetical model of B-cell/MP interactions in B-cell lymphomas. This model predicts that indolent and retransformed or non-transformed lymphoma cells do not initially exhibit autonomous or spontaneous-independent neoplastic cell growth but instead must undergo interactions with indigenous non-neoplastic cells, specifically, monocytes or macrophages (MP)-derived tumor-associated MPs (TAMs) that bind, stimulate, and activate targeted lymphoma cells, to adopt aggressive autonomous phenotypes. The postulated mechanism for this relationship is that a high monocyte count is a surrogate biomarker for the tumor microenvironment, reflecting the functions of immunosuppressive peripheral blood monocytes recruited by lymphoma cells to differentiate monocytes into polarized MPs (M2) that can in turn activate the tumor cells. In addition, the aggregated TAMs (red) can protect tumor cells and, potentially, cancer stem cells (blue) within their clusters from chemotherapy, enabling the tumor cells to survive (residual disease) and re-establish (relapse) at a later time.

Our preliminary data demonstrated that MPs that formed in culture (*in vitro*) co-existed with MCL cells for extended periods (>2 months), after which mitotic figures in the MPs began to appear (Figure 2). These results also suggested that these lymphoma-derived MPs are not normal, unlike non-mitotic normal MPs. How do these MCL-associated MPs differ from normal MPs? Our preliminary data also indicated that when we purified the MPs in the latter stages of tissue culture (3–4 months), some of them phenotypically expressed PAX5 (a B-cell marker) and genotypically had the t(11;14) translocation (unpublished data). A possible explanation for this phenomenon is that at some point during tissue culture, polarized M2 MPs fuse with MCL cells, creating hybrid cells that may divide and/or proliferate. MP/cancer cell fusion is not uncommon, as recent studies demonstrated that these hybrid cells may spontaneously acquire cancer stem cell properties, including enhanced drug resistance, self-renewal, and increased metastatic activity (105–107).

### B-Cell-to-MP Reprogramming

Another mechanism of the derivation of MPs in B-cell lymphomas may be lineage switching or reprogramming from B cells to myeloid cells, as certain subsets of B cells are endowed with self-renewal capacity and the ability to adopt gene expression patterns, morphology, and functional activities characteristic of MPs (108, 109). Studies performed by Xie et al. (110) provided strong evidence that committed mature B-cell-lineage cells can be reprogrammed to become MPs. Using a retroviral approach, they induced overexpression of the transcription factor C/EBPβ in CD19+ murine bone marrow B-cell progenitors. As a result, about 60% of the B cells had downregulated expression of B-cell-restricted genes (e.g., CD19, Rag1, B220, Mb-1, EBF, and Pax5) and upregulated expression of MP-restricted genes (e.g., MAC-1, Fc gamma RIII, Fc gamma R1, M-CSF-R, and PU.1). These reprogrammed B cells not only had MP phenotypes but also adopted the morphology of MPs and gained phagocytic abilities and/or functions. Apparently, introduction or switching of a single transcription factor can set into motion an entire series of events that can transform a B cell into a macrophagic cell. Whether this occurs naturally or only under certain pathological conditions, such as cancer, remains unclear, particularly in humans. In a recent article, McClellan and colleagues reported that when incubated *in vitro*, blasts from some precursor B-cell acute lymphoblastic leukemia cells could apparently be functionally reprogrammed into myeloid lineage-mimicking cells that morphologically resemble and can, in some cases, function similarly to normal MPs (111). Several other studies had similar findings, demonstrating that malignant B-lymphoid cells can be reprogrammed to become MPs with apparently lymphoid and myeloid characteristics under certain conditions (112–115). Interestingly, we observed a similar phenomenon in MCL cases, as *in vitro* culture of primary MCL cells led to the presence of MPs after several days (70). Whether such a reprogramming mechanism exists in MCLs must be confirmed to determine whether this mechanism can be used for therapeutic purposes.

## Functional Significance of TAMs in the B-Cell Lymphoma Microenvironment

Normal human MPs are typically ubiquitous, polymorphous, usually large, heterogeneous, multifunctional, myeloid-derived regulatory cells. These cells are active in most tissues and organs, mediating a wide array of biological regulatory activities to at least some extent in most normal eukaryotic tissues. Alternatively, putative neoplastic counterpart MPs (TAMs) are described as having both protumor and antitumor properties, but the majority of clinical lymphoid cancer studies have demonstrated that the presence of a high number of TAMs in the TME is related to poor prognosis, suggesting that TAMs predominantly exert protumoral activity *via* various mechanisms in many B-cell lymphomas.

### Stimulation of Tumor Cell Growth and Survival

Normal MPs function mainly by engulfing foreign substances such as cellular debris and microbes as well as moribund and apoptotic cancer cells *via* phagocytosis. On the other hand, TAMs are usually large, polarized, multifunctional cells with great cellular plasticity that can play multiple key activating roles in the initiation and progression of many tumor types (116). TAMs are also prime candidates for creating the microenvironmental milieu, histologically represented by pathologists, particularly in MCL patients, as pink histiocytes, but they are present within tumor tissues in much greater numbers than once thought after immunohistochemical analysis identified them *via* staining of human tissue biopsy samples for anti-CD68 monoclonal antibodies. Many of these tumors frequently appear in tissue areas exhibiting chronic inflammation, likely aided by the mutagenic actions of MPs. Tumor growth and progression are often supported by MP-induced survival and stromal cell production *via* various MP-produced tumor-stimulating growth factors (117). Our *in vitro* investigations using primary MCL cell cultures demonstrated that MCL cells usually died within 2 weeks of *in vitro* culture if TAMs were not present, indicating or at least suggesting that TAMs are required for maintenance of the viability of primary MCL cells in long-term *in vitro* cell culture. The dependence of malignant B cells on some types of myeloid cells for continued survival is not unprecedented, as CD5+ B1-derived chronic lymphocytic leukemia (CLL) cells are highly dependent on nurse-like cells with M2 TAM phenotypes for mediation of growth and survival (118–120). However, whether the interaction between tumor cells and TAMs actually requires cell–cell interactions or can occur through a paracrine secretory mechanism has yet to be determined. In either case, coculture of TAMs with various types of lymphoma cells can activate various intrinsic pathways, such as signal transducer and activator of transcription 3, phosphoinositide 3-kinase/mammalian target of rapamycin, and nuclear factor-κB, providing key growth and survival signals in tumor cells (121–123).

### Provision of Chemoresistance Mechanisms to Cancer Cells

Cancer cells at both primary and secondary metastatic tumor sites become resistant to various chemotherapeutic drugs through various molecular mechanisms mediating the activity of intrinsic and/or extrinsic cellular factors, although the latter can often remain largely overlooked. In most tumors, a high density and accumulations of TAMs predict poor outcomes, and TAMs are highly present in relapsed and refractory lymphomas, most likely playing an important role in multiple types of drug resistance. Mounting evidence suggests that the TME also play critical roles in multiple aspects of tumor progression, particularly in therapeutic resistance (124). The lymphoma microenvironment can be composed of several different subsets of host cells, in particular, bone marrow stromal cells and TAMs, where they may play a key role in B-cell survival that to promote drug resistance (125). TAMs are often key components of the TME and often play an important role in the biology of different types of lymphoma, including FL, HL, DLBCL, and MCL (70, 126). The potential role of TAMs in B-cell lymphomagenesis and the emerging field of lymphoma B cell–TAM interaction should be further investigated to determine whether these two cellular subsets undergo bidirectional cross talk in the context of lymphoma drug resistance. TAM-mediated drug resistance can be a form of *de novo* drug resistance that can protect tumor cells from the initial effects of diverse therapies *via* both soluble factor-mediated and cell adhesion-mediated drug resistance. One hypothesis explaining this mechanism is that specific niches within the lymphoma microenvironment provide sanctuaries for subpopulations of tumor cells and a survival advantage through host-cell/tumor-cell interactions, activating key pathways that allow the target cells to survive the insult of toxic therapy, resulting in minimal residual disease (Figure 2). Over time, residual tumor cells are destined to expand and evolve through acquisition of additional genetic abnormalities (or selection of preexisting clones of tumor cells) that cause the gradual development of more complex, diverse, long-standing acquired resistance phenotypes. Persistence of residual tumor cells eventually cause relapsed disease, which is much less likely to respond to subsequent therapy after acquired resistance develops and the disease ultimately progresses. Therefore, understanding the relationship between host and tumor cells may help in the identification as well as design of more effective therapies to overcome dissemination or recurrence of cancer and improve the ultimate outcomes of future cancer therapies.

B lymphocytes are divided into two subpopulations—B1 and B2 cells—based mostly on expression of the definitive T-cell-associated protein CD5 (127). Natural B1 cells are further divided into B1a cells, which express CD5 on their membranes, and B1b cells, which do not express CD5 but share most other biological characteristics of B1a cells. CD5+ B1-cell origins and the predisposition of these cells toward giving rise to lymphoma and leukemia are long-standing issues that have yet to be successfully addressed experimentally (128). B1 (CD5+ B) cells appear early in ontogeny, produce mainly unmutated polyreactive antibodies, and are capable of self-renewal. B1 cells clonally expand as they age and are the primary malignant tumor cells in B-cell CLL and MCL cases. B1 cells are also immunogenic, capable of secreting inflammatory chemokines and cytokines. Our experimental focus in MCL studies has been on better characterizing the MCL microenvironment containing candidate endogenous MCL stem-like cell components. Several recent reports supported such a stem cell-like concept in human MCL cell populations with specific immunophenotypes (e.g., CD45+ CD19, CD133+ CD19−) (129, 130). However, whether individual clones of tumor-initiating stem cells within the MCL TME niche-expressing population of MCL cells remain stable over time and are relatively resistant to conventional MCL therapies remains unknown. TME niche-expressing MCL cells may be composed of cells that survive over the long term in tumors and give rise to daughter cells that can maintain the tumor’s existence over time. Alternatively, they may be pluripotent and represent different clones, each having tumor-initiating cell characteristics at different points in time. Within the normal bone marrow microenvironment, MPs are crucial for the maintenance of normal hematopoiesis of stem cells (131, 132). TAMs may play a similar role in MCL cases, maintaining the stem-like cellular phenotype within the TME niche in the bone marrow as stable disease (indolent), with the MCL progressing to and/or transforming (blastoid) into more aggressive disease at a later time.

### Immunosuppression

Tumor-associated MPs may also alter the behavior of the human cellular immune system, impacting the efficacy of recently developed immune checkpoint inhibitors (PD-1, PD-L1, etc.) and affecting T lymphocytes, leading to disease progression (133–136). Some malignant B cells acquire intrinsic mechanisms to escape from immune surveillance by tumor-specific cytotoxic T cells *via* overexpression of PD-L1 or PD-L2 on the cell surface or recruitment of TAMs expressing PD-L1. These ligands interact with the PD-1 receptor expressed on intratumoral T cells and provide an inhibitory signal, thereby suppressing the antitumor immune response. Checkpoint inhibitors, such as the anti-PD-1 antibody, bind to the checkpoint receptor PD-1 expressed on T cells, stimulating their proliferative capacity and enabling the immune system to reactivate its ability to recognize, attack, and destroy cancer cells. Several anti-PD-1/PD-L1 regimens have had encouraging therapeutic effects in patients with relapsed or refractory HL, FL, or DLBCL (137). Although immune checkpoint inhibitors are emerging as promising therapeutic options for patients suffering from different types of cancer, including aggressive B-cell lymphomas, the challenge is that not all treated cancer patients have had responses to these checkpoint inhibitors. Understanding the mechanisms that control PD-1/PD-L1 expression in various types of cancer cells and the key involved accessory cells may not only identify important predictive biomarkers for controlling the efficacy of anti-PD-1/PD-L1 antibody-based immunotherapy but also help in the development of novel targeted therapies that can be combined with checkpoint inhibitors for additional clinical efficacy. Although researchers have well established that PD-1 and/or PD-L1 blockade activates important immune T cells, little is known about the mechanistic role that the PD-1/PD-L1 pathway may play in TAMs. Several studies have focused on the expression of PD-L1 in TAMs and demonstrated that numerous well-known signaling pathways, such as the key oncogenic pathways MYC and signal transducer and activator of transcription 3, are responsible for PD-L1 regulation (98, 138, 139).

## Targeting TAMs in B-Cell Lymphoma Patients

Tumor-associated MPs, particularly those with the M2 phenotype, are among the major constituents of the tumor stroma in patients with different types of cancer, including B-cell lymphomas. Also, investigators have obtained compelling preclinical as well as clinical evidence that TAMs can promote neoplastic initiation, malignant progression, and further metastasis. TAMs are therefore potential targets for adjuvant therapies for aggressive B-cell lymphoma, particularly relapsed or refractory lymphomas. Strategies designed to deplete TAMs or inhibit their recruitment into neoplastic lesions have been successful in experimental settings and are now considered promising therapeutic approaches in the clinic. To therapeutically target TAMs, researchers have proposed using various pharmacological as well as immunological strategies described below based on the results of previous preclinical studies.

### Clodronate

Clodronate is a first-generation bisphosphonate-family compound that is now used in the clinic to prevent or actively inhibit the development of bone metastases and treat inflammatory diseases such as autoimmune rheumatoid arthritis and osteoarthritis (140). With experimental encapsulation of clodronate into liposomes, researchers developed an efficient reagent that selectively depleted MPs when successfully applied in several immunological studies (141). Recently, investigators found that the use of bisphosphonates as antiangiogenic agents suppressed tumor growth as well as metastasis in several lymphoma models, including DLBCL and T-cell lymphoma models, primarily through elimination of MPs (142).

### The Bruton’s Tyrosine Kinase Inhibitor Ibrutinib

Ibrutinib, a novel, first-in-class, orally bioavailable, irreversible Bruton’s tyrosine kinase inhibitor, recently exhibited clinical effectiveness and tolerability in clinical trials in patients with various hematological malignancies, including refractory CLL and MCL (143, 144). However, whether ibrutinib actually inhibits the biological activity of MPs remains unclear. A recent study demonstrated that ibrutinib could actually target cells of monocyte/MP lineage in autoimmune disease models (145). In addition, clinical data indicated that ibrutinib-based treatment in MCL patients led to decreased secretion of MP inflammatory proteins and chemokines into the plasma (146). In the bone marrow microenvironment in CLL patients, ibrutinib disaggregated the interactions of MPs with leukemia cells by inhibiting secretion of the chemokine CXCL13, which decreased the chemoattraction of CLL cells (147). These findings support the concept that ibrutinib can target MPs in patients with B-cell lymphoma/leukemia and function *via* this chemokine/cytokine type of mechanism.

### Trabectedin

Trabectedin is an antitumor chemotherapeutic drug originally isolated from a marine organism and approved by the U.S. Food and Drug Administration for the treatment of sarcoma and ovarian carcinoma (148, 149). Recently, researchers showed that trabectedin has selective cytotoxicity in cells of myeloid lineages, particularly TAMs, through the mechanism through induction of caspase-dependent apoptosis (150). Evidence in both mice and sarcoma patients suggests that selective MP depletion is a key mechanism of action mediating the antitumor activity of this agent (151). In biopsy samples obtained from sarcoma patients given trabectedin, authors noted a marked decrease in the number of TAMs and related stroma vessel networks (152). Trabectedin has undergone evaluation in several different *in vitro* and *in vivo* cancer models, exhibiting similar activities by targeting and selectively eliminating tumor-associated monocytes and MPs (153). Taking the acceptable toxicity profile of trabectedin and its unusual mode of action into account, this compound is an important therapeutic agent because it interferes with not only tumor cells but also myeloid cells in the TME. These results provide proof of principle for MP targeting in cancer patients and may have implications for the design of additional combination therapies, particularly for various relapsed and/or refractory B-cell lymphomas.

### CSF-1/1R Inhibitors

CSF-1/1R signaling is a key survival signaling pathway in MPs, as blocking this signaling pathway preferentially depletes M2-like MPs while sparing M1-like MPs (154). CSF-1R signaling blockade can be achieved using antibodies against CSF-1/CSF-1R or other small-molecule inhibitors (155, 156). Anti-CSF-1R monoclonal antibodies are currently under preclinical and clinical evaluation for treatment of various solid tumors, indicating the possibility of using these antibodies against lymphoid malignancies (157, 158). Small-molecule CSF-1R kinase inhibitors such as pexidartinib (PLX3397) have effectively reduced the numbers of TAMs in several different cancer models (158–162). For example, deletion of TAMs by pexidartinib enhanced antitumor immunity and survival induced by immunotherapy in patients with solid tumors (160).

## Concluding Remarks

B-lymphoid cells represent one of two essential components of the human cellular immune system that continue to be linked with MPs under normal, pathological, and neoplastic conditions. For more than 50 years, MPs have been recognized as relatively independent multifunctional hematological and immunological cells with only limited or tangential interrelationships with each other. Many studies have revealed that individualized biological and/or immunological functions of MPs represent wide functional flexibility, making them capable of recognition of and/or interaction with a wide variety of normal as well as neoplastic immune cells, although B-cell tumors at times have converted or transformed into monocytes/MPs or even have possibly been capable of actually becoming biphenotypic. In fact, researchers have shown that MPs and B cells have almost limitless cellular interactive potential, particularly in the immune and inflammatory systems, but are by no means limited to these essential areas. With the advent of increased realization and recognition of important aspects of modern molecular genetics along with the role and extended research capabilities, these multifunctional, seemingly limitlessly flexible cell types continue to play even larger and more complex roles in newly recognized forms of contemporary immunotherapy and cellular immunology. In fact, MPs and B cells, which are two of the most important primary mammalian immune/inflammatory cell types, are responsible for maintaining many elements of human immune system. These immune cell components are derived, at least in part, from the quite close relationships of components of myeloid/MP and B-lymphoid cell lineages that provide key functional cellular and humoral immune response components with multiple intercellular linkages to the complex, intricate human immune system.

In the innate immune system, TAMs (M2, polarized, alternatively activated MPs), which can play multiple critical wide-ranging roles in basic biological activities, often mediate enhanced tumor progression in patients with poor clinical prognosis, demonstrating multiple functional pathophysiological capabilities. These include the ability to secrete key chemokines, cytokines, and various bioactive proteases that have been shown capable of stimulating tumor cell growth, angiogenesis, metastasis, chemoresistance, and immunosuppression. Of particular interest and possible importance, some recent studies demonstrated that M2-polarized MPs can express key immunotherapeutic targets, such as checkpoint proteins (e.g., PD-1) that appear to be involved in T-cell activation, as well as targets of other specific checkpoint-blocking immunotherapies (anti-PD-1/PDL-1) currently of interest as part of new therapeutic paradigms for understanding the conceptual basis for chemotherapy-resistant neoplasms.

Whereas much is known about the wide spectrum of plasticity and cellular flexibility of MPs in many normal and pathological cellular settings, relatively little is known about the increasingly important interactions between MPs and B-lymphoid cells, particularly in the TMEs of patients with aggressive forms of NHL-B. Our own studies have defined what we believe to be the necessary and sufficient conditions for establishing better *in vitro* models for defining the actual mechanisms driving the pathophysiology of B-cell lymphoma cells. Preliminary studies demonstrated that efficient *in vitro* NHL-B cell growth not only requires the presence of viable clonal NHL-B cells but also often requires the presence of large, autochthonous monocytes/MPs and probably other related myeloid-derived cells that are necessary to maintain lymphoma cell growth with adequate cellular viability to yield persistent, effective cell-line establishment. Such findings have proven to be essential for developing new effective therapeutic strategies, particularly for relapsed/refractory NHL-B, by targeting TAMs with monoclonal antibodies (anti-CSF-1R) or small-molecule inhibitors of CSF-1R kinase (PLX3397).

Cumulative studies over the past 50 years have brought to light the critical role of TAM/B-cell interactions in the pathophysiology of NHL-B. Now is the time to use this important and practical knowledge to further develop new novel strategies to better treat this deadly disease, as more than 30% (~20,000) of NHL-B patients die every year of disease processes that we should be able to reverse if not actually cure with a better understanding of the pathophysiology and capabilities demonstrated using improved model systems established in recent years.

## Author Contributions

All authors listed have made a substantial, direct, and intellectual contribution to the work and approved it for publication.

## Conflict of Interest Statement

The authors declare that the research was conducted in the absence of any commercial or financial relationships that could be construed as a potential conflict of interest. The reviewer TM and the handling Editor declared their shared affiliation.
